# Structure/Function Analysis of PARP-1 in Oxidative and Nitrosative Stress-Induced Monomeric ADPR Formation

**DOI:** 10.1371/journal.pone.0006339

**Published:** 2009-07-29

**Authors:** Ben Buelow, Burak Uzunparmak, Marcia Paddock, Andrew M. Scharenberg

**Affiliations:** 1 Departments of Pediatrics and Immunology, University of Washington, Seattle, Washington, United States of America; 2 Division of Immunology, Seattle Children's Hospital Research Institute, Seattle, Washington, United States of America; University of Hong Kong, Hong Kong

## Abstract

Poly adenosine diphosphate-ribose polymerase-1 (PARP-1) is a multifunctional enzyme that is involved in two major cellular responses to oxidative and nitrosative (O/N) stress: detection and response to DNA damage via formation of protein-bound poly adenosine diphosphate-ribose (PAR), and formation of the soluble 2^nd^ messenger monomeric adenosine diphosphate-ribose (mADPR). Previous studies have delineated specific roles for several of PARP-1′s structural domains in the context of its involvement in a DNA damage response. However, little is known about the relationship between the mechanisms through which PARP-1 participates in DNA damage detection/response and those involved in the generation of monomeric ADPR. To better understand the relationship between these events, we undertook a structure/function analysis of PARP-1 via reconstitution of PARP-1 deficient DT40 cells with PARP-1 variants deficient in catalysis, DNA binding, auto-PARylation, and PARP-1′s BRCT protein interaction domain. Analysis of responses of the respective reconstituted cells to a model O/N stressor indicated that PARP-1 catalytic activity, DNA binding, and auto-PARylation are required for PARP-dependent mADPR formation, but that BRCT-mediated interactions are dispensable. As the BRCT domain is required for PARP-dependent recruitment of XRCC1 to sites of DNA damage, these results suggest that DNA repair and monomeric ADPR 2^nd^ messenger generation are parallel mechanisms through which PARP-1 modulates cellular responses to O/N stress.

## Introduction

Converging evidence from pharmacologic and genetic studies suggests that the poly adenosine diphosphate-ribose polymerases PARP-1 and PARP-2 play a central role in cellular responses to environmental oxidative and nitrosative (O/N) stress [1]. Two major pathways appear to lie downstream of PARP-1/2 activation: formation of nuclear polymeric adenosine diphosphate-ribose (PAR) associated with the cellular response to oxidant-induced DNA damage (reviewed in [1], see also [2]–[4]), and formation of monomeric adenosine diphosphate-ribose (mADPR) that serves as a 2^nd^ messenger to induce gating of the TRPM2 Ca^2+^ channel [5]–[8].

A detailed model for PARP-1 function in the context of O/N stress-induced DNA damage has emerged in which PARP-1 is activated by binding of its N-terminal domain (designated the DNA binding domain or DBD) to oxidant-induced DNA single strand breaks (SSB) and double strand breaks (DSB) [9]. Activated and DNA bound PARP-1 catalyzes the conversion of cellular nicotine adenine dinucleotide (NAD) to long, branched chains of PAR attached to a wide variety of acceptor proteins in the nucleus. Notably, the major PAR acceptor is PARP-1 itself, which appears to accumulate roughly 90% of cellular PAR via PARylation of its auto-modification domain (AMD) [1]. DNA bound PARylated PARP-1 and associated proteins are thought to promote relaxation of the 30 nm chromatin fiber and destabilization of DNA-histone interactions to allow additional DNA damage response proteins access to the damaged site [10]. In the case of DNA SSBs, the combined actions of PAR-ylated PARP-1 and the PARP-1 BRCT domain contribute to the assembly of a protein complex at the break site that includes XRCC1, DNA Ligase III and DNA pol-β [11]–[16]. In the case of DSBs, PAR/PARP-1 are thought to promote homologous recombination-mediated repair (HR) through the recruitment and PARylation of factors involved in non-homologous end joining (NHEJ) including Ku70 and DNA-PKcs, resulting in the inhibition of their ability to bind free DNA ends [17]–[20].

**Figure 1:**
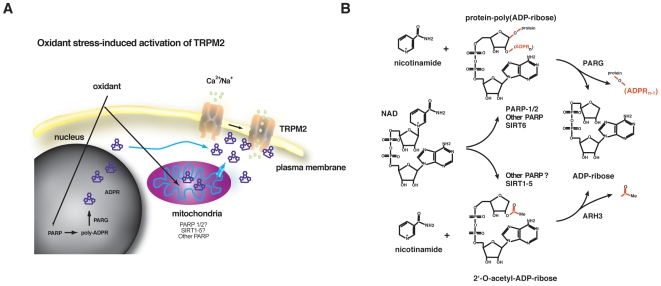
PARP-1 synthesizes long, branched chains of poly-mADPR (PAR) on a wide variety of acceptor proteins in the nucleus, notably PARP-1 itself (which appears to accumulate roughly 90% of cellular PAR). Subsequently, PAR is degraded by Poly-ADP-Ribose-Glycosylase (PARG) into mADPR, which is assumed to diffuse out of the nucleus and into the cytosol. There it can bind to the ion channel TRPM2, leading to the influx of cations, including calcium. Additionally, PARP-1 mediates the recruitment of DNA repair proteins to sites of DNA damage through protein-protein interactions (notably through its BRCT domain) and PARylation. The relationship between PARP-mediated mADPR degradation and DNA damage repair remains unclear.

**Figure 2:**
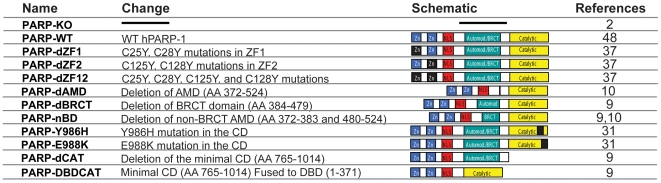
human polyADP-ribose polymerase-1 (PARP-1) variants. PARP-1 variants with mutations or deletions of each of hPARP-1′s functional domains were made based on previous successful expression of the mutant construct either in bacteria or eukaryotic cells. Zn – zinc finger domain. Automod – automodification domain. BRCT – BRCA1 C-terminal homology domain. Catalytic – PARP catalytic domain.

Much less is known about the biochemical mechanisms of PARP-1 activation in the context of O/N stress induced formation of mADPR. Compelling evidence indicates that PARP-1-dependent mADPR formation results in mADPR-mediated activation of the TRPM2 Ca^2+^ channel (Figure 1 and [21]–[24]). However, there are no data addressing the biochemical context in which PARP-1 activation leads to mADPR formation, or the relationship between these mechanisms and PARP-1′s involvement in the DNA damage response. To better define the biochemistry of PARP-dependent mADPR formation, we reconstituted PARP-1 deficient DT40 cells with either WT or various mutant forms of PARP-1 (Figure 2), and determined the capacity of each mutant to support two correlates of O/N stress-induced mADPR formation: NAD degradation and TRPM2 activation. Our results suggest that catalytic activity, DNA binding, and an intact auto-PARylation domain are required for PARP-1-mediated cytosolic mADPR accumulation *in vivo*. In contrast, the BRCT domain, which is required for recruitment of the DNA repair factor XRCC1, was found to be entirely dispensable. Taken together, these results suggest that DNA repair complex formation and mADPR 2^nd^ messenger accumulation are parallel mechanisms through which activated PARP-1 signals the presence and extent of O/N stress to a cell.

## Results

### Expression of PARP-1 structural mutants in DT40 B lymphocytes

To dissect the contributions of each of PARP-1′s domains to mADPR accumulation under conditions of O/N stress *in vivo*, we generated a series of human PARP-1 mutants that had been previously successfully expressed and characterized *in vitro* (Figure 2 and legend).

**Figure 3:**
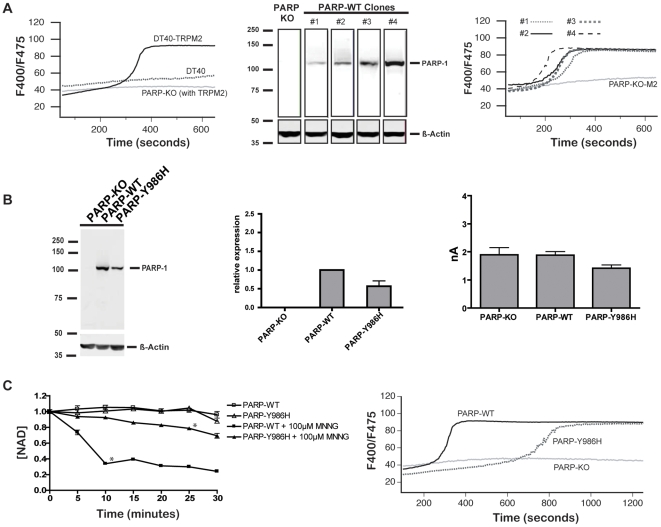
Attenuation of PARP-1 catalytic activity correlates with a reduction in O/N stress induced NAD degradation and TRPM2-dependent Ca^2+^ transients. *A) Left panel*: Calcium transients in DT40 cells are PARP and TRPM2 dependent. The indicated cell lines were stimulated with 100 mM MNNG, and cytosolic Ca^2+^ transients were monitored by ratiometric analysis of Indo-1 fluorescence by FACS. *Middle panel*: PARP clones with varying PARP expression levels by western blotting: PARP-deficient DT40 cells were reconstituted with WT human PARP-1, and the parent PARP-KO line and four clones with a range of expression levels are shown. 50 µg of cellular protein was loaded into each lane of an 8% SDS-PAGE gel and analyzed by western blotting. Rabbit anti-human PARP-1 polyclonal antibody was used as the primary antibody for immunoblotting of PARP-1 (1∶4000, Alexis Biochemicals), and IR680 conjugated goat anti-rabbit as secondary antibody (1∶3000, Licor Inc). Blots were analyzed on a Licor Odyssey. *Right panel*: Dependence of TRPM2-dependent Ca^2+^ transients on PARP expression level. The four PARP-WT expressing clones from the middle panel were compared for TRPM2-dependent Ca^2+^ transients in response to 100 µM MNNG, as measured by ratiometric analysis of Indo-1 fluorescence by FACS. Time to half maximum F400/F475 showed a standard deviation of±33 seconds: clone #2 with an intermediate level of PARP-1 expression was designated as *PARP-WT* and used as a positive control for subsequent experiments. *B) Left Panel*: *PARP-Y986H* protein expression is comparable to *PARP-WT*: 50 µg of cellular protein were loaded into each lane of an 8% SDS-PAGE gel and analyzed by western blotting. Antibodies were identical to *A.) Middle Panel*: Relative transcript abundance of *PARP-Y986H* normalized to *PARP-WT*, as determined by Q-PCR. *Right panel*: TRPM2-dependent whole cell currents are similar in *PARP-WT* and *PARP-Y986H* mutant clones. Average whole cell currents were not statistically different from one another across all cell types. Cells were patched in the whole cell configuration: the pipette solution contained 100 µM mADPR. The I-V relationship and current development across all cell types was characteristic of TRPM2 and identical to that previously shown by our lab (4). At least 3 whole cell recordings were taken for each cell type. *C) Left panel*: NAD turnover in *PARP-WT* and *PARP-Y986H* cells. Stars indicate a p-value of p≤.001 from baseline for all subsequent points. *Right panel*: TRPM2-dependent Ca^2+^ transients in *PARP-WT* and *PARP-Y986H* cells after stimulation with 100 µM MNNG, as measured by ratiometric analysis of Indo-1 fluorescence by FACS.

Because direct measurement of cellular mADPR is confounded by the degradation of NAD and/or NADP into mADPR during nucleotide extraction procedures (reviewed in [25]–[27]), our experimental approach utilized two indirect readouts of each mutant PARP's ability to support mADPR formation: NAD degradation and TRPM2-dependent cytosolic Ca^2+^ transients. Degradation of NAD is the sole metabolic pathway leading to the formation of mADPR, and thus its degradation is directly correlated with the rate and magnitude of mADPR formation [28]–[30]. As discussed above, TRPM2 is a Ca^2+^ entry channel that is the physiologic target of free cytosolic mADPR, and mADPR-medated TRPM2 activation results in characteristic short latency, large magnitude cytosolic Ca^2+^ transients [5], [6], [31]. Although cytosolic Ca^2+^ can be influenced by any factor that affects Ca^2+^ entry to or exit from the cytosol, in the DT40 system, large magnitude O/N stress-induced Ca^2+^ transients require the expression of TRPM2 as well as PARP-dependent formation of free cytosolic mADPR (Figure 3A, left panel and (30)). Thus, these characteristic Ca^2+^ transients can be used as a surrogate marker of cytosolic mADPR accumulation to the TRPM2 gating threshold.

To allow correlated assessment of NAD degradation and TRPM2-dependent Ca^2+^ transients in a single experimental system, a TRPM2 expressing PARP-deficient DT40 B lymphocyte line was chosen as a testbed for the mutant PARPs. To evaluate the influence of hPARP expression on TRPM2-dependent Ca^2+^ responses, PARP-KO cells were transfected with WT human PARP-1 to generate a panel of clonal cell lines with a range of hPARP-1 expression (Figure 3A, middle panel). Over the range of hPARP-1 expression observed in these clones, only small differences in latency were observed in TRPM2 dependent Ca^2+^ transients induced by the model nitrosative stressor MNNG (Figure 3A – right panel: time to half maximum F400/F475 = 327±33 sec.), suggesting that across this range of hPARP-1 expression levels, PARP-1 activity is not limiting for accumulation of free cytosolic mADPR to a level required to initiate TRPM2 gating. Clone #2, with intermediate expression of hPARP-1, was designated *PARP-WT* and used as a positive control for subsequent experiments.

Following transfection of TRPM2 expressing PARP-deficient DT40 cells with each mutant construct (Figure 2), panels of clones were selected and TRPM2 and mutant PARP protein expression were compared to the *PARP-WT* control cell line selected above. For each PARP mutant, a clone closely matched to the *PARP-WT* cell line for both hPARP-1 by western blot (Figures 3B [left panel], 4A [left panel], and 5A [left panel]) and TRPM2 by whole cell current amplitude (Figure 3B [right panel], 4A [right panel], and 5A [right panel]), was selected for use in subsequent experiments. Importantly, taken together with the data from Figure 3A, these data demonstrate that, absent a significant alteration in function of a mutant protein relative to WT PARP-1, the range of expression levels observed in the clones chosen for analysis would not be expected to significantly impact the rate of O/N-stress induced mADPR accumulation.

**Figure 4:**
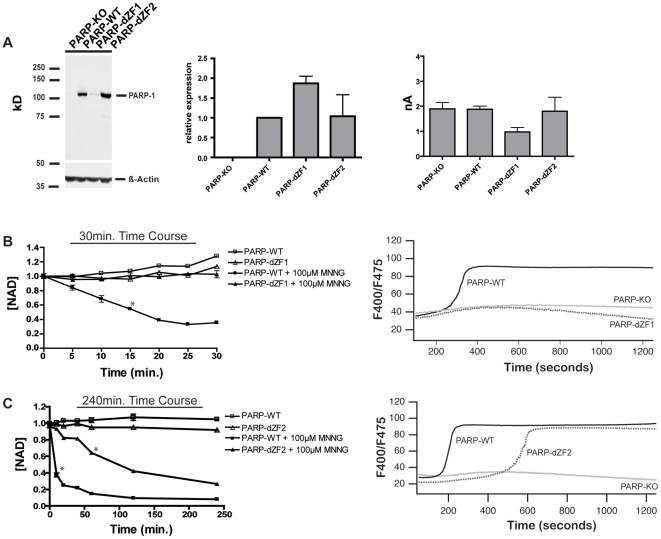
The effects of DBD mutations on PARP-dependent O/N STRESS induced NAD degradation and TRPM2-dependent Ca^2+^ transients. *A) PARP-dZF* mutant expression levels relative to PARP-WT:*Left Panel*: 50 µg of cellular protein were loaded into each lane of an 8% SDS-PAGE gel and analyzed by western blotting. Rabbit anti-human PARP-1 polyclonal antibody was used as the primary antibody for immunoblotting of PARP-1 (1∶4000, Alexis Biochemicals), and IR680 conjugated goat anti-rabbit as secondary antibody (1∶3000, Licor Inc). Blots were analyzed on a Licor Odyssey. *Middle Panel*: Relative transcript abundance of *PARP-dZF* mutants normalized to *PARP-WT*, as determined by Q-PCR. *Right panel*: TRPM2-dependent whole cell currents are similar in *PARP-WT* and *PARP-dZF* mutant clones. Average whole cell currents were not statistically different from one another across all cell types. Cells were patched in the whole cell configuration: the pipette solution contained 100 µM mADPR. The I-V relationship and current development across all cell types was characteristic of TRPM2 and identical to that previously shown by our lab [5]. At least 3 whole cell recordings were taken for each cell type. *B) Left panel*: NAD turnover in *PARP-WT and PARP-dZF1* DT40 cells. *PARP-dZF1* did not show NAD degradation over the course of 30 minutes following application of 100 µM MNNG. Stars indicate a p-value of p≤.001 from baseline for all subsequent points. *Right panel*: TRPM2-dependent Ca^2+^ transients in *PARP-WT and PARP-dZF1* DT40 cells after stimulation with 500 µM MNNG, as measured by ratiometric analysis of Indo-1 stained cells by FACS. No transients were seen in *PARP-KO* or *PARP-dZF1* at 100 µM MNNG (data not shown). *C) Left panel*: NAD turnover in *PARP-WT and PARP-dZF2* DT40 cells. *PARP-dZF2* cells show NAD degradation over the course of 240 minutes following application of 100 µM MNNG. Stars indicate a p-value of p≤.001 from baseline for all subsequent points. *Right panel*: TRPM2-dependent Ca^2+^ transients in *PARP-WT and PARP-dZF2* DT40 cells after stimulation with 100 µM MNNG, as measured by ratiometric analysis of Indo-1 stained cells by FACS. No transients were seen in *PARP-KO* or *PARP-dZF2* at 100 µM MNNG (data not shown).

**Figure 5:**
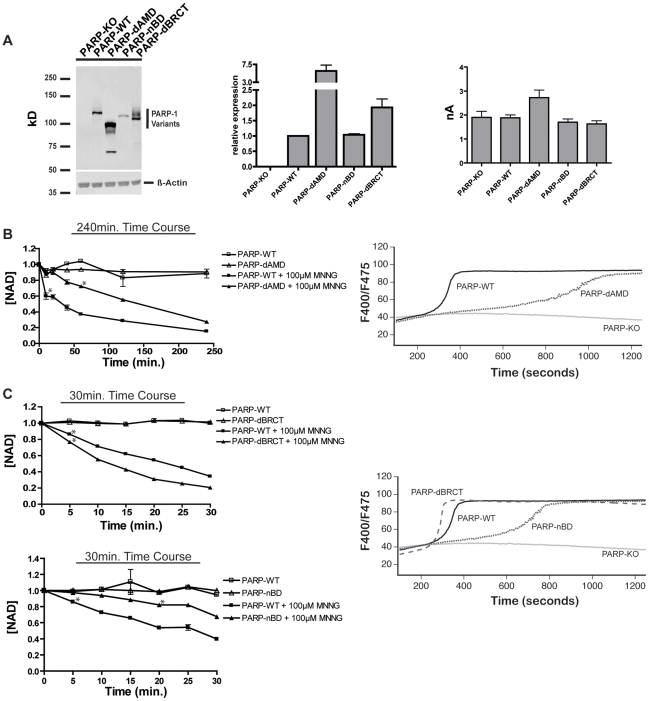
Loss of the AMD domain, but not the BRCT domain, is associated with reduced NAD degradation and TRPM2-dependent Ca^2+^ transients. *A) Left Panel*: *PARP* AMD mutant expression levels relative to PARP-WT 50 µg of cellular protein from each cell line were loaded into each lane of an 8% SDS-PAGE gel and analyzed by western blotting. Rabbit anti-human PARP-1 polyclonal antibody was used as the primary antibody for immunoblotting of PARP-1 (1∶4000, Alexis Biochemicals), and IR680 conjugated goat anti-rabbit as secondary antibody (1∶3000, Licor Inc). Blots were analyzed on a Licor Odyssey. *Middle Panel*: Relative transcript abundance for PARP AMD mutants normalized to *PARP-WT*, as determined by Q-PCR. *Right Panel* TRPM2 dependent whole cell currents are similar in *PARP-WT* and PARP AMD mutant clones. Average whole cell currents were not statistically different from one another across all cell types. Cells were patched in the whole cell configuration: the pipette solution contained 100 µM mADPR. The I-V relationship and current development across all cell types was characteristic of TRPM2 and identical to that previously [5]. At least 3 whole cell recordings were taken for each cell type. *B) Left Panel*: NAD turnover in *PARP-WT* and *PARP-dAMD* cells. Stars indicate a p-value of p≤.001 from baseline for all subsequent points. *Right Panel*: TRPM2-dependent Ca^2+^ transients in *PARP-WT* and *PARP-dAMD* cells after stimulation with 100 µM MNNG, as measured by ratiometric analysis of Indo-1 stained cells by FACS. *C) Left Panels*: NAD turnover in *PARP-WT*, *PARP-dBRCT*, and *PARP-nBD* cells. Stars indicate a p-value of p≤.001 from baseline for all subsequent points. *Right Panel*: TRPM2-dependent Ca^2+^ transients in *PARP-WT*, *PARP-dBRCT*, and *PARP-nBD* cells after stimulation with 100 µM MNNG, as measured by ratiometric analysis of Indo-1 stained cells by FACS.

### Catalytic activity correlates with PARP-1 NAD degradation and mADPR accumulation under conditions of O/N stress

*In vitro*, a functional PARP-1 catalytic domain is known to be required for PAR synthesis. *In vivo*, TRPM2 activation (a surrogate of mADPR accumulation, as described above) occurs early in an O/N stress exposure time course, with onset of gating occurring at time points when c.a. 15–20% of cellular NAD is degraded [5]. In order to validate the role of PARP-1 catalytic activity in O/N stress induced NAD degradation and TRPM2 gating, we generated a panel of catalytic domain (CD) mutants with varying levels of in vitro PARP activity [9], [32]: hPARP-1 lacking the catalytic domain (*PARP-dCAT*), hPARP1 DBD fused directly to the minimal catalytic domain (*PARP-DBDCAT* – this mutant can bind DNA but is catalytically inactive, [33], [34], [Kupper, 1996 #99,35]], hPARP-1 with ∼99% attenuated catalytic activity *in vitro* (*PARP-E988K*), or hPARP-1 with ∼90% attenuated catalytic activity *in vitro* (*PARP-Y986H*) (see Figure 2). For functional evaluation, cell lines expressing each construct (as indicated by their acquired resistance to hygromycin treatment following transfection) were evaluated for NAD degradation and their ability to support a TRPM2 dependent Ca^2+^ transient (Figure 3B). Surprisingly, although abundant transcript was detectable, and these proteins have been reported as stable *in vitro* and in other cell systems [9], [36], the *PARP-E988K* and *PARP-dCAT* mutants failed to express in DT40 cells, suggesting a loss of protein stability in the DT40 context. The inactive PARP-DBDCAT, behaved as predicted, expressing as well as WT PARP but exhibiting no capacity to support a TRPM2-dependent Ca^2+^ transient (data not shown). Consistent with *in vitro* results suggesting that its activity is about 90% reduced from WT PARP-1, the *PARP-Y986H* mutant resulted in both a detectable but significantly delayed time course of NAD degradation, and an increased latency TRPM2-dependent Ca^2+^ transient (Figure 3C, left and right panels). Based on the data shown in Figure 3A, the slightly decreased expression level of the *PARP-Y986H* mutant would not be expected, in isolation, to reduce the rate of mADPR accumulation sufficiently to cause the observed changes. Taken together, these results directly implicate PARP-1 catalytic activity in the formation and accumulation of mADPR.

### Zinc Finger mutations of the PARP-1 DBD significantly compromise both NAD degradation and mADPR accumulation under conditions of O/N stress

PARP-1 has two predicted zinc finger motifs in an N-terminal region that has been implicated in DNA-binding dependent activation of PARP-1, and that is designated the DNA binding domain (DBD). Previous work *in vitro* and *in vivo* has suggested that mutation of the first zinc finger (ZF1) in PARP-1′s DBD leads to loss of DNA binding and PARP activation in response to both DNA SSBs and DSBs, while mutation of the 2^nd^ zinc finger (ZF2) leads to a relatively selective loss of response to SSB's [34], [36], [37]. Though there is some evidence that the catalytic domain of PARP has a very limited activity independent of the DBD [33], neither *in vivo* NAD degradation nor mADPR accumulation were examined in previous studies which addressed DBD function.

To evaluate the role of the DBD in PARP-dependent mADPR formation *in vivo*, we studied cells expressing PARP-1 in which two cysteines in either ZF1 (*PARP-dZF1*), ZF2 (*PARP-dZF2*), or both ZF1 and ZF2 (*PARP-dZF12*) were replaced with tyrosine residues, thereby disrupting the structure of the targeted zinc finger(s). Q-PCR analysis revealed that transcript levels of the two single mutants *PARP-dZF1* and *PARP-dZF2* (Figure 4A) were within 1-2 fold those of the PARP-WT control line. However, *PARP-dZF1* showed considerably less expression than *PARP-dZF2*, suggesting a loss of stability and/or degradation of this mutant. Supporting the importance of structurally intact zinc fingers to PARP folding *in vivo*, *PARP-dZF12* showed no protein expression in DT40 cells in any screened clones (data not shown).

If DNA binding were necessary for PARP-dependent mADPR accumulation, the *PARP-dZF1* mutant, which has little or no detectable DNA binding capacity, would be expected to show little or no ability to reconstitute O/N stress-induced NAD degradation or TRPM2 activation in the DT40 system. Consistent with this prediction, we observed no detectable reconstitution of NAD degradation and TRPM2 dependent Ca^2+^ transients with the dZF1 mutant (Figure 4B, left and right panels). While the low expression level of PARP-*dZF1* prevents the unequivocal conclusion that the dZF1 mutant lacks functional activity due to loss of DNA binding, results from other PARP mutants with comparable relative expression but retained functional reconstitution (e.g. see Figure 5, PARP-nBD mutant) suggest that if dZF1 had retained a significant level of activity, some functional reconstitution would be expected at the expression level that was achieved.

Using similar reasoning, as the ZF2 mutant retains DNA binding activity towards SSBs, one would expect attenuated NAD degradation and TRPM2-dependent Ca^2+^ transients, particularly at lower levels of O/N stress. Consistent with this prediction, treatment of *PARP-dZF2* expressing cells with 100 µM MNNG led to NAD degradation, but over a prolonged time course in comparison to PARP-WT cells (Figure 4C, left panel), while the generation of a TRPM2-dependent Ca^2+^ transient required treatment with 500 µM MNNG (Figure 4C, right panel, no response was observed with 100 µM MNNG treatment over a 1200 second period of time, data not shown).

Overall, our analyses of the dZF1 and dZF2 mutants suggest that PARP-recruitment to sites of DNA damage via the DBD is crucial to both NAD degradation and mADPR mediated TRPM2 gating. They also support previous *in vitro* data on the relative importance of each ZF domain in PARP activation: ZF1 function is required for PARP activation, and ZF1 alone is able to sustain a reduced level of PARP activation and PARP-dependent NAD degradation and cytosolic mADPR accumulation when ZF2 is inactivated.

### The BRCT- and non-BRCT- components of the PARP-1 AMD contribute differentially to O/N stress induced NAD degradation and mADPR accumulation

Previous work has shown that PARP-1 interacts with a wide variety of proteins through its BRCT domain or PAR moieties [15], [38]–[40]. Both the BRCT domain and the primary sites of PAR moiety attachment are found in a region of PARP-1 known as the automodification domain (AMD). In order to evaluate the contribution of protein interactions mediated via the AMD domain to mADPR accumulation, a PARP mutant lacking the entire AMD domain, *PARP-dAMD*, was generated. Although a highly overexpressing clone was used for analysis (Figure 5A), PARP-deficient DT40 cells reconstituted with the *PARP-dAMD* mutant showed a significantly decreased rate of NAD degradation (Figure 5B, left panel) and a significantly increased latency of TRPM2 dependent Ca^2+^ transients under conditions of O/N stress (Figure 5B, right panel), suggesting a significant compromise of mADPR formation.

The observed compromised mADPR formation could be due either to functional inactivation of PARP-1 catalytic activity on account of the large deletion, loss of PARylation sites resulting in decreased converstion of PAR to mADPR, or loss of protein interactions required for generation of mADPR. The BRCT domain of the AMD contains numerous possible automodification sites (9/15 glutamates found in the AMD are in the BRCT domain) and has been implicated in protein-protein interactions of PARP-1, including its homodimerization (which is thought to be important for catalytic activity and automodification [41]), recruitment of XRCC1, and recruitment of PARG (necessary for PAR turnover [10]). A plausible explanation for the compromised NAD degradation and TRPM2 gating associated with the PARP-dAMD mutant is therefore that PARP-dAMD is unable to recruit appropriate effector proteins via the BRCT domain. To test this possibility, we examined PARP-deficient DT40 cells expressing either of two additional mutant PARP constructs: *PARP-dBRCT*, which lacks only the BRCT portion of the AMD, and *PARP-nBD*, which retains only the BRCT domain and has all other elements of the AMD deleted (see Figure 1). Remarkably, *PARP-dBRCT*, which expressed at the same level as PARP-WT (Figure 5A), fully reconstituted O/N stress dependent NAD degradation (Figure 5C, top left panel), and TRPM2 dependent Ca^2+^ transients (Figure 5C, right panel). Although *PARP-nBD* was expressed at lower level than WT PARP in clone #2, it was still able to partially reconstitute NAD degradation (Figure 5C, bottom left panel), and support a TRPM2 dependent Ca^2+^ transient, albeit with a prolonged latency (Figure 5C, right panel). Taken together, these findings suggest that the AMD has an important role in PARP-1 mediated mADPR accumulation, but that this role is independent of protein interactions mediated by the BRCT domain.

## Discussion

Although the biochemical mechanisms through which PARP-1/2 are activated to induce PAR synthesis in the context of the DNA damage response have been established both *in vitro* and *in vivo*, the specifics of how their activation leads to mADPR accumulation remain largely untested. By reconstituting PARP-1 deficient DT40 cells with either WT or variously mutated PARP-1 isoforms, we here provide *in vivo* data which define the contribution of PARP-1′s major domains to O/N stress induced cytosolic mADPR accumulation. Taken together, our results demonstrate a requirement for PARP-1 catalytic activity, DNA binding capability, and auto-PARylation for PARP-induced mADPR accumulation, and indicate that protein interactions mediated via the BRCT domain are dispensable for this function.

It has previously been shown that PARP-1 accumulation at sites of DNA damage *in vivo* is dependent on the integrity of ZF1 [37]. *In vitro* studies have suggested that ZF1 is required for PARP-1 activation in the presence of both SSBs and DSBs, while ZF2 makes only a minor contribution to activation by SSBs. Our results support the hypothesis that PARP-mediated mADPR accumulation is initiated by the same or very similar processes, as we observed that ZF1 is absolutely required for PARP-1 mediated mADPR accumulation, while ZF2 makes an important, albeit smaller, contribution. Given that *PARP-dZF2* cannot bind DSBs yet shows undiminished activation by SSBs in vitro, our results further suggest that in response to MNNG, PARP-1 mediated NAD degradation and mADPR accumulation may be occurring largely in response to SSBs, as MNNG induces primarily SSBs [42], [43]. In the absence of a functional ZF2, a higher concentration of MNNG is able to detectably activate PARP, an observation which may reflect a sufficiently high density of SSBs or sufficient numbers of DSB's to induce the activation of PARP via ZF1 alone.

Following binding of damaged DNA and activation of PARP-1, PARP-1 mediates PAR accumulation on itself and other proteins as well as the formation of a DNA repair complex. Previous data [9], [10] have suggested that the BRCT domain is involved in the formation of the DNA repair complex, but have not addressed whether DNA repair complex formation is associated with mADPR 2^nd^ messenger formation. Our results demonstrate that BRCT-dependent interactions are not required for PAR/mADPR accumulation, suggesting that DNA repair complex formation and mADPR generation functions are at least parallel, and possibly independent. Overall, our results extend previous *in vitro* results indicating that loss of the BRCT domain does not influence PARP-1 activity [9], and that interactions mediated by the BRCT domain are not required for mADPR formation.

Early work on PARP-1 suggested that the AMD is the exclusive target of PARP-1 automodification. 15 glutamates are present in the AMD, representing roughly 50% of the total possible auto-modification targets in PARP-1: of these, 9 are in the BRCT portion [44]–[47], and the other 6 in the nBD protion. Nevertheless, *PARP-dBRCT* showed no apparent defect in NAD degradation or induction of TRPM2 dependent Ca^2+^ transients. Since the decrease in our surrogate markers of PARP-1 dependent mADPR accumulation was much more dramatic for *PARP-dAMD* than for *PARP-dBRCT*, our results further suggest that PARP-1 may automodify specific glutamates in the nBD regions preferentially. Alternatively, recently described specific lysine ADP-ribosylation in the AMD may account for our observations [33]. Although evidently not absolutely required for mADPR accumulation, such preferential ADP-ribosylation of specific amino acid residues could have a role in other aspects of PARP function.

Taken together, our results suggest that the biochemical mechanisms required for PARP-1′s role in O/N stress induced ADPR formation are very similar or identical to those involved in PARP-1′s participation in the response to DNA damage. ZF1 and ZF2 work in concert to mediate activation of PARP-1 via binding to oxidant-induced SSBs and DSB's. Following DNA binding and activation, PARP-1 PARylates itself within the non-BRCT portions of the AMD domain, as well as other proteins. At this point PARP-1′s downstream functional paths appear to diverge, as the BRCT domain together with some portion of protein-bound PAR mediates interactions to assemble a DNA repair complex as a component of PARP-1′s function in the DNA damage response, while other PAR is apparently degraded by PARG into mADPR, which diffuses into the cytosol resulting in TRPM2 gating and a Ca^2+^ transient.

## Materials and Methods

*Cell Culture*—Wild type DT40 B lymphocytes (DT40 control cells) and DT40 B lymphocytes constitutively expressing wild type TRPM2 (DT40-TRPM2 cell line) were cultured at 37°C with 5% CO_2_ in RPMI 1640 medium (Mediatech Inc.) supplemented with 10% fetal bovine serum (Mediatech Inc.), 2% chicken serum (Invitrogen), 10 units/ml penicillin/streptomycin (Mediatech Inc.), 2 mM glutamine (Mediatech Inc.), and β-mercaptoethanol (50 µM; Sigma). PARP-1-deficient DT40 cells were a generous gift from Shunichi Takeda.

*O/N stress stimulations*— Previously, we have shown that MNNG and H_2_O_2_ elicit very similar PARP-1 dependent NAD degradation and TRPM2 dependent Ca^2+^ transients: because MNNG produces more consistent responses, we chose to use it for all experiments.

*Molecular Biology*—PARP-deficient cells expressing HA-TRPM2 were previously generated in our lab [5]. PARP constructs were generated by having subsections of PARP-1 synthesized (Blue Heron, Inc.) and then cloning these fragments into the pcatalytic domainNA5TO-hPARP-1 vector previously described [5]: all constructs were sequenced in their entirety. Stable transfection of PARP-deficient DT40 B lymphocytes with the various human PARP-1 constructs (see Figure 2: hPARP-1 cDNA courtesy of Ted Dawson, The Johns Hopkins University Medical School, [48]) was carried out using a Bio-Rad Gene-Pulser electroporation apparatus. Cells (1×10^7^/0.5 ml of serum-free medium) were pulsed in 0.4-cm cuvettes with 50 µg of plasmid DNA at 550 V and 25 µF. Clones were selected in hygromycin (2 mg/ml, Calbiochem). Individual clones were evaluated for hPARP-1 by western blot and TRPM2 expression via whole cell patch clamp (see below) and western blot.

*Calcium Imaging*—Changes in cytosolic calcium concentration were measured by quantitating changes in the ratio of blue/violet fluorescence (in arbitrary units) from the dye Indo-1 (Molecular Probes). Briefly, 1×10^7^cells in 1 mL of media were incubated with Indo-1 dye (final concentration: 7 µg/ml) for 45 minutes in the dark at 37°C. Cells were washed twice with 1xPBS (Cellgro) and resuspended in 1 mL RPMI 1640 medium (as described above) without phenol red indicator dye. Cells were analyzed for 30 seconds on an LSR II (Becton Dickinson) to establish a baseline, treated with either 1 or 5 uL 100 mM MNNG (final concentration: 100 or 500 µM), and analyzed for a total of 1200 seconds. All recordings were done at room temperature.

*Whole Cell Patch Clamp*—DT40 cells were adhered to glass coverslips by a 10-min incubation in serum-free media and then stored in standard media (see above) until transferred to the extracellular solution (145 mM NaCl, 2.8 mM KCl, 10 mM CsCl, 1 mM CaCl_2_, 2 mM MgCl_2_, 10 mM glucose, 10 mM HEPES, pH 7.4). Patch clamp experiments were carried out using a Zeiss Axiovert microscope, equipped with Eppendorf micromanipulators and stage control. Patch clamp recordings were taken using an EPC10 amplifier (Heka) and a Dell computer running Pulse. Cells were patched with borosilicate glass pipettes pulled with a Sutter instruments P-97 micropipette puller: pipettes had 3–8 MΩ tips. Pipettes were filled with intracellular solution containing 145 mM cesium glutamate, 8 mM NaCl, 1 mM MgCl_2_, and 10 mM HEPES, pH 7.4, with or without 100 µM mADPR. Following formation of a gigaseal, the membrane patch was ruptured by suction, and recordings were taken for 100–300 s. 50-ms voltage ramps were run every 2 s from −100 to +100 mV. Data analysis was performed using Pulse and Excel software.

*NAD Assay*—The NAD assay was performed according to the method of Jacobson and Jacobson (reviewed in [27]) with minor modifications. Briefly, 1×10^6^ cells were resuspended in 290 µl of media in a single well of a 96-well plate. Wells received 10 µl of 3 mM MNNG (for a final concentration of 100 µM) at 5-min intervals over a total time course of 30 or 240 min (three replicates per time point). Cells were pelleted at 1500 rpm for 5 min and lysed in 100 µl of 0.5 N HClO_4_ for 15 min on ice and in the dark. Cellular debris was removed by centrifugation at 1500 x *g* for 10 min. On ice and in the dark, 100 µl of KOH and 100 µl of K_2_HPO_4_/KH_2_PO_4_, pH 7.2, were added to the supernatant and incubated for 15 min. The KClO_4_ precipitate was removed via centrifugation at 1500 x *g* for 10 min. 25 µl of the supernatant was incubated for 5 min at 37°C with 2 mM phenazine ethosulfate, 0.5 mM 3-(4,5-dimethylthiazol-2-yl)-2,5-diphenyltetrazolium bromide, 50 mM EDTA, 600 mM ethanol, and 120 mM Bicine, pH 7.8. 12.5 µl of alcohol dehydrogenase (1 mg/ml, Sigma) were added, and the plate was incubated for a further 20 min at 37°C. The plate was then read (absorbance) for 1 s/well using a VICTOR3 plate reader at 570 nm. Data analysis was performed in Microsoft Excel and GraphPad Prism. Fluctuations among untreated series from all cell lines between 80 and 120% of base line were observed, which were judged to lack practical relevance. To control for these, a conservative statistical significance level of *p*<0.001 was chosen, as deviations from base line in untreated controls in individual plates did not reach statistical significance by this criterion.

*Western Blotting*—Western blotting was performed in 8% acrylamide (29∶1) SDS-polyacrylamide gels. Lysis of cells was performed in RIPA lysis buffer with complete mini protease inhibitor cocktail (Roche). Debris was removed by centrifugation at 20,000 x *g* for 15 minutes at 4°C, and protein in the supernatant was quantified using a bradford protein assay (BioRad). Immunoprecipitation of HA-TRPM2 was done on 500 µg of protein from relevant cell lines incubated with HA antibody (1∶100, Cell Signaling) overnight and precipitated using protein A beads. Nuclear/Cytoplasmic extraction was performed as follows: Cells were vortexed 3 times in Buffer S10 (10 mM HEPES, pH7.9, 10 mM KCl, and 0.1 mM EDTA) and centrifuged at 20,000 x *g* for 10 minutes at 4°C. The resulting pellet was then vortexed 3 times in Buffer S100 (20 mM HEPES, pH7.9, 500 mM NaCl, 1 mM EDTA, and 10% glycerol) and centrifuged at 20,000 x *g* for 10 minutes at 4°C. Protein in each of the two supernatants was quantified using a Bradford protein assay (BioRad). The following primary antibodies were used: monoclonal anti-HA (1∶1000, Cell Signaling), monoclonal anti-β-actin (1∶40,000, Sigma) and polyclonal rabbit anti-human PARP-1 (1∶4000, Alexis Biochemicals). Secondary antibodies were IR680 conjugated goat anti-rabbit (1∶3000) and IR800 conjugated goat anti-mouse (1∶10,000) from Licor and Invitrogen, respectively. Visualization was performed using a Licor Odyssey. Note that in Figure 3A, clones 1–4 were run on a single gel, along with several other redundant clones. Individual lanes were separated and displayed to illustrate the range of hPARP-1 expression across which PARP-1 expression confidently does not alter mADPR accumulation as measured by TRPM2 dependent Ca^2+^ transients.

*Q-PCR*: catalytic domainNA was prepared using the Qiagen RNAeasy kit. Q-PCR was performed on a BioRad icycler using the BioRad SYBR green master mix. The annealing temperature was 58°C. The following primers were used: hu/ck Parp F: gagtacgccaagagcgggc, hu/ck Parp R: atgggcgactgcaccatg, Beta Actin F: tgagagggaaatcgtgcgtgacatc, Beta Actin R: caggaaagagggttggaacagagcc. PARP-1 transcript level was normalized to β-actin and *PARP-WT* expression using the ΔΔCt method. Data analysis was performed using Microsoft Excel and Graphpad Prism.
